# 

**Published:** 1807-12

**Authors:** 


### NEW MEDICAL PUBLICATIONS.

A Practical Treatise on the Prevention and Cure of the Venereal Disease, by T. M'Caton. 8vo, 2s.

A Dictionary of Chemistry and Mineralogy; with an account of the process employed in many of the most important Chemical Manufactories, with Plates, &c. by A. and C. R. Aikin. 2 vol. 4to. 3l. 13s. 6d. boards.

Chemical Chatechism, by S. Parkes. 12s. boards.

---

END OF VOL. XVIII.

### INDEX.

A.

ABDOMINAL vifcera, on difeafes of the 19
Abernethy, Mr. on the digeftive organs 21
Adams, Dr. on empiricifm 3
——— his defence of vaccination 28
——— on morbid poifons 82
Affectation in medicine, on 253
Africa, account of vaccination in 147
African Negro, cafe of gout in an 578
Air, on vitiated 288
Albinos, defcription of an 9
Alder, Dr. on contagion and fever 423
———, Dr. on quarantines 497
Aliquis, in anfwer to T. Y. 160
Althœa, its medical properties 458
America, account of antiquities in 483
——— ftate of medicine 40
Amicus, on chronic rheumatifm 430
Ammonia, its ufe in narcotic poifons 19
Andrews, Dr. on ftrictures in the urethra 380
Aneurifm, on a cafe of popliteal 158
——— cafe of doubtful 299
——— on the treatment of 375
——— cafe of popliteal 475
——— cafe of femoral 508
Animal bodies, on peculiarities in the functions of 36
Ankle-joints, compound fracture of 162
Anus, cafe of imperforate 297
Aorta, cafe of ruptured 391
Arabia, ravages of the fmall-pox in 192
Arfenic, its effects in rheumatifm 277
——— in fungus hæmatodes 389
——— in hydrophobia 401
———, on its ufe in rheumatifm 565
Arthritic cafes, ufe of horfe-radifh in 273
Affizes, particulars of the black 95
Afthenia, cafes of 188
Afthma, remedy in pituitous 271
——— on convulfive 476
Atmofphere, on infectious 86

B.

Baildon's Dr. account of a Chinefe remedy 176
Balmis Dr. account of his medical voyage 37
Banks, Sir J. meeting of the faculty at 4
———, on the treatment of tender plants 386
Barclay, Dr. on ophthalmia 15
Bardfley's Dr. medical reports of cafes 275
Bellamy's Dr. cafe of calculus in the urethra 319
——— cafe of difeafed bladder 511
Bell's anatomy of expreffion, account of 2
Bellott's Mr. cafe of hernia 45
Birth, account of a monftrous 344
Bifhopp's Mr. cafe of fracture of the fternum 415
Bifmuth, ufes of the oxyd of 281
Bladder, cafe of difeafed 511
Blane, Dr. on yellow fever 468
Bleeding from fmall veffels, how to ftop 199
Blindnefs by fmall-pox, on 428
Blifters, their ufe in checking mortification 443
Blood-letting, its efficacy in a wound of the lungs 333
——— in difficult parturition 337
——— in hydrophobia 402
BOOKS, CRITICAL ANALYSIS OF NEW MEDICAL.—Enquiry into the Seat and Nature of Fever, by Henry Clutterbuck, M. D. 74. Obfervations on Morbid Poifons, by Jofeph Adams, M. D. 82. The Edinburgh Medical and Surgical Journal, No. XI, 175. Differtations on Man, by J. Jarrold, M. D. 178. Obfervations on Medical Reform, 185. Medical Reports of Cafes and Experiments, with Obfervations chiefly derived from Hofpital Practice, by S. A. Bardfley, M.D. 275. Practical Obfervations on the Uterine Hæmorrhage, by John Burns, 281. Obfervations on the Application of Lunar Cauftic to Strictures in the Urethra and Œfophagus, by M. W. Andrews, M. D. 380. Pethox Parvus, dedicated without Permiffion, to the Blind Priefts of that Idolatry, by Iconoclaftes, 384. The Ancient and Modern Hiftory of Nice, by J. B. Davies, M. D. 385. A Letter, containing Obfervations on Fractures of the Lower Limbs, by Sir James Earle, *ib.* A Rowland for an Oliver, by John Ring, 386. An Effay on the Nature of Fever, by A. P. Wilfon, M. D. 462. The Edinburgh Medi-

Q q cal and Surgical Journal, No. XII, 468, 577. A Practical Inquiry into Disordered Respiration, by Robert Bree, M. D. 476. Chemical Philosophy, by W. by A. F. Fourcroy, translated by Desmond, Esq. 573.

BOOKS, NEW MEDICAL, ANNOUNCED. The second volume of the Botanist's Guide, in Northumberland and Durham, 95. Dr. Miller's edition of Williams's Mineral Kingdom, *ib.*—Turnbull's System of British and French Surgery, 96, 292. Parkes's new edition of the Chemical Catechism, 192. Dr. Keglegoot, on Medical Jurisprudence, 193. Dr. Young, on Medical Knowledge, *ib.* Dr. Cheyne, on Hydrocephalus Acutus, *ib.* Dr. Barton's Elements of Zoology, 194. Mott, on the Statice Lemonium of Linnæus, *ib.* Hofack's Catalogue of Plants, *ib.* Report of the College of Physicians, 292. Dr. Reid's Treatise on Consumption, *ib.* Dr. Beddoes, on Fever, *ib.* Second Part of the Medical Observer, *ib.* Dr. Halliday, on Emphyzema, *ib.* Dr. Alibert, on Disorders of the Skin, 388. Accum's System of Mineralogy, *ib.* Young's Lectures on Surgery, 434. Mr. Sadler's Encyclopædia of Manufactures, 579. Dr. Trotter, on the Nervous Temperament, 580. Mr. Wishart's Translation of Scarpa, on Aneurism, *ib.* Dr. Roberton's Natural History of the Atmosphere, *ib.* Dr. Saunders, on Pulmonary Consumption, *ib.* The same, on Hydrocephalus, *ib.* Mr. Wardrop, on the Pathology of the Eye, *ib.* Mr. Parkinson's Organic Remains of the former World, *ib.* Mr. Prouss, on Insanity, *ib.* Mr. Wilson's Ornithology of America, *ib.*

Botanical description of British plants, 169, 270, 363, 457

Bougies, on metallic 243

——— on tobacco 351

Bourne, Dr. on the uva ursi 9

Bowels, on disorders of the 372

Bozzini's light spreader, described 39

——— experiments with 387

Brain, on a demonstration of the 157

——— cases of wounded 359

——— fever, case of 154

Breast, Chinese remedy for the 176

Bree, Dr. on disordered respiration 476

Brown's Dr. theory, observations on 464

Budd, Dr. on the effects of corrosive sublimate 347

Burn, case of brain fever succeeding a 152

Burns, Mr. on the gravid uterus 22

——— on the uterine hæmorrhage 281

——— on the treatment of 149, 206, 238, 360, 405

——— case of 304

C.

Cabanis on the revolutions of medical science 35

Cabbage, varieties and uses of the 367

Caldwell, Dr. on quarantines 111

Cancer, on carbonate of iron in 16

——— observations on 534

Cantharides, on the internal uses of 21

——— efficacy in glanders, &c. 556

Carbonic gas in mortification, effects of, 536

Cataleptic paroxysm, case of 37

Caustic, on lunar 380

Cayley, Dr. answer to 48

Ceylon, on the mineralogy of 483

Chalmers, Mr. answers to 56, 61

Charlatanism, on 3

Child, resuscitation of a still-born 307

China, state of vaccination in 29

Chin-cough, on the 566

Cholera, cases of 373

——— prevalence of 287

Chorea sancti viti, on 213

Cinchona, strange remedy for the 34

Clutterbuck, Dr. on the seat and nature of fever 74

Cold affusion recommended in hydrophobia 400

Coley's, Mr. case of tuberculated liver 485

Consumption, observations on 11

——— effects of labour in pulmonary 448

Contagion, on 423

Controversies, on medical 256, 420

Convulsions, new method of treating 94

——— case of puerperal 353

Copenhagen, success of vaccination at 28

Correspondents, to 96, 196, 292, 484

Corrosive sublimate, disease produced by taking 347

Cotta's, Dr. history of quackery, character of 3

Cough, case of violent 54

——— observations on 91

——— benefits of erysimum in 364

Coxe, Dr. on chorea sancti viti 213

——— his case of burn successfully treated 304

——— case of tinea capitis 340

——— case of tetanus 436

——— case of a wound of the intestines, 501

Crito, answers to 481 432

——— his reply 374

Cuming, Dr. on refrigeration in fever, 197

Cutaneous eruptions, remedy in 171

D.

Davies, Mr. H. on medical controversies 256 537

——— replies to 315, 420

Dawes, Mr. on the economy of the liver, 293

——— remarks on 426

Dawson's, Mr. case of popliteal aneurism 475

Delirium, case of 152

Denmark, Mr. in reply to Mr. Chalmers 61

Derbyshire Practitioner, his reply 48, 309, 432

——— observations in answer to, 156, 374

Dewees, Dr. on blood-letting in rigidity of the os externum 337

——— his examination of Dr. Osborn's opinion 450 545

——— Mr. Merriman's remarks on 417

Diabetes mellitus, cases and observations on 273

Diarrhœa, on the treatment of 566

Digestive organs, on diseases of the 21

Digitalis, observations on the use of 176

——— in hydrophobia, on 401

Diseases, on hereditary 182

——— in London, account of 91, 93, 187, 285, 287, 371, 373, 482, 486, 572

Dog, account of the opening of the abdomen of a living 294

Dorsey's, Dr. case of wounded brain 359

Drinking, case of paralysis, from suddenly leaving off 155

Dropsy, useful remedy in 273

——— remedy for 171

Dublin, report of the College of Physicians of 103

——— Pharmacopœia, observations on the 540

Dumas, M. on peculiarities in human bodies 36

E.

Ear, account of the anatomy of the 5

——— case of worms in the 379

Earle, Sir J. on fractures of the lower limbs 385

Earnest, Mr. on burns and scalds 405

Edinburgh, reports of the Colleges of Physicians and Surgeons of 105

——— Medical and Surgical Journal reviewed 175, 468, 577

——— medical degrees conferred by the university of 190

——— report of diseases in 565

Edmonston, Dr. on ophthalmia 12

Egyptian ophthalmia, observations on the 12, 474

Electricity in hydrophobia, on 401

——— theory of galvanic 245

Electricity, case of fever from rheumatism relieved by 362

Elephantiasis, on the 84

Elmer, Dr. on mortification of the uterus 344

Empiricism, observations on 3

Empyema, case of 392

Epidemic, on the Gibraltar 8

Epilepsy, observations on 17

——— benefits of cardamine in 272

——— efficacy of sugar of lead in 355

——— of digitalis in 172

Eruptions, remedy in cutaneous 171

Erysinum, its use in coughs 364

Estremadura, analysis of ore found in 193

Evans's, Mr. case of fracture 162

——— Dr. cases of burns 208

Exanthemata, on the 90

Exeter, account of the black assize at 89

Expression, on anatomical 8

Eye, on diseases of the 16

F.

Farcy, on the cure of the 555

Fever, on the seat and nature of 74

——— case of brain 153

——— on refrigeration in 177

——— from rheumatism 362

——— on contagion and 423

——— on the nature of 462

Ffirth, Dr. on hepatitis 301

Fibula, case of fracture of the 309

——— case of imperforate 346

Flux, remedy for bloody 169

Fluxes, use of the geum urbanum in 436

Fœtus, cases of small-pox in the 177

Fomites, observations on 13, 88

Forbes's, Mr. cases of small-pox in the fœtus 177

Fothergill's, Dr. account of diseases in London 93, 182, 285, 371, 482, 569

Fowler's mineral solution, efficacy of 250

Fox, Mr. on stopping decayed teeth 553

Fracture, case of, successfully treated 129

——— of the ankle joints, case of 162

——— Mr. Warnock's case of 309

France, on the means of promoting medical science in 35

——— population of cities in 96

——— proposals for remunerating Dr. Jenner in 341

Fraser's, Dr. essay on epilepsy, account of 17

Freake, Mr. on the humulus lupulus 403

Frydenberg, Dr. on the feetang 191

Fungus hæmatodes, cases of 389

——— observations on 534

G.

Gall, Dr. on the fyftem of 38
Galvanic electricity, new theory of 245
Galvanifm in paralyfis, effects of 279
Gaftritis, on a cafe of 61
Gaftrodynia, cafe of 163
George's, St hofpital, lectures at 387
Geum urbanum, ufes of the 486
Gibraltar epidemic, on the 8
Glanders, new mode of treating the 555
Glafgow, lectures in the univerfity of 289
Gleet, cafe of obftinate 155
Golding, Mr. reply to 136
Gorcy's, M. hiftory of hydrophobia 475
Gout, on the treatment of 52, 163, 240
—— moft fevere in cold climates 183
—— in an African Negro, cafe of 578
Gravid uterus, on the 22
Greafe, obftinate, in horfes, mode of treating 555
Green, Mr. on a fatal cafe of hydrocele 531
Greenfield, Dr. on the ufe of cantharides 21

H.

Hæmorrhage, on the uterine 281
———— remedy for 174
———— from the lungs, on blood-letting in 320
Hands, on treating paralyfis of the 21
Harrifon, Dr. on medical reform 4
———— cafe of hydrophobia 236
Heidman's, Dr. theory of galvanic electricity 245
Hemoptyfis, cafe of 320
Hepatitis, on the ufe of nitric acid in 301
Hereditary difeafes, on 182
Hernia, cafe of incarcerated femoral 45
——— account of focieties for relief of 33
Herpes pauperum, on the 87
Hicks, Mr. on hydrophobia 258
Hiftorical fketch of the progrefs of medicine 1
Holland, medical jurifprudence in 193
Home's Dr cafe, obfervations on 77
Horfe-radifh, virtues of 272
Horfes, mode of treating diforders in 555
Huggan, Dr. on hydrophobia 396
Humulus lupulus, on the 403
Hunt's, Mr. anatomical reflections 7
Hunter's mufeum, appropriation of 386
Hydrocele, remarks on a cafe of 531
Hydrocephalus internus, cafes of 12, 481
Hydrophobia, Dr. Harrifon's cafe of 236
———— Mr. Hicks on 258
———— Dr. Huggan on 396
———— hiftory of 475
———— Dr. Mofeley's cafe of 550
Hymen, obfervations on the 37

I. J.

Iceland, account of a valuable plant in 191
Inoculation, report of the College of Phyficians on vaccine 97
———— obfervations on 150
———— on fmall pox 384
Inftitutions, on charitable 192
Inteftines, extraordinary cure of a wound of the 501
Introfufception, cafe of 175
———— Dr. Shaw's hiftory of a cafe of 350
Itch, obfervations on the 84
Jackfon, Dr. on the Gibraltar epidemic 8
Jail fever, account of the 89
Jarrold's, Dr. differtations on man 178
Jaundice, remedy for the 170
Jenkinfon, Mr. on the mineral folution of Fowler 250
Jennings, Dr. on labour in pulmonary confumption 448
Jenner, Dr. parliamentary grant to 32
———— propofal in France for remunerating 341
Johnfon's, Mr. cafe of fever from rheumatifm relieved by electricity 362
Joints, cafe of fracture of the 162
Jouville's, M. obfervations in Ceylon 483
Juniper, virtues of 370

K.

Kentifh's, Dr. treatment of burns 412
Kermes, on the procefs for obtaining 35
Kinglake's, Dr. treatment of gout, obfervations on 52, 163
———— on the treatment of burns and fcalds 206
———— reply to Mr. Pope 240
Kitfon's, Mr. cafe of fchirrhous ulceration 475

L.

Labour, its effects in pulmonary confumption 448

Langftaff's, Mr. cafe of introfufception 175

Lafkey, Mr. on the London Mufeum 434

Lazarettos, obfervations on 114

Lead, fugar of, its efficacy in epilepfy 355

Lectures—Mr. Walker on phyfiology, 194. At the univerfity of Glafgow, 289. At St. Thomas's and Guy's hofpitals, 290. Theatre London hofpital, *ib.* Mr. Brookes on anatomy, phyfiology, and furgery, *ib.* Mr. Wilfon on anatomy, phyfiology, pathology, and furgery, 291. Mr. Accum on operative chemiftry and mineralogy, *ib.* Dr. Badham on the practice of physic, chemistry, and the materia medica, *ib.* Dr. Bradley on the theory and practice of medicine, *ib* Mr. Carlisle on the art and practice of surgery, *ib* Mr. Carpue on anatomy and surgery, *ib.* Dr. Clarke and Mr. Clarke on midwifery and the diseases of women and children, *ib.* Dr. Dennison and Squire on the theory and practice of midwifery, *ib.* Dr. Marshall on midwifery, *ib.* Mr. Moor on the teeth, 292, 388. Mr. Thomas on surgery, *ib.* At St. George's hospital, 387. Dr. Clough on midwifery, *ib.* Dr. Hooper on the theory and practice of physic, *ib.* Dr. Reid on the theory and practice of medicine, 388. Mr. Taunton on anatomy, physiology, pathology, and surgery, *ib.*

Legs, a singular affection of the 278

Leprosy, observations on 182

Libraries, proposals for village 194

Light spreader, account of the 39, 387

Limbs, on fractures of the lower 385

Liver, on the economy of the 293, 426

—— case of ruptured 391

—— case of an enlarged and tuberculated 485

London Hospital, lectures at the 290

—— Museum, on the 434

Lunar caustic, on 380

Lungs, effects of blood-letting in an hæmorrhage from the 320

—— in a wound of the 333

Lusus naturæ, account of a 319

M.

M'Gregor, Dr. on the ophthalmia 474

M'Whirter's, Dr. case of delirium 152

Madness, observations on 182

Mallow, marsh, its virtues 458

Mammoth, enormous tooth of the 95

Marseilles, on the plague at 119

Marson, Mr. on vaccination 70

—— answer to 150

Martin's, Mr. case of imperforate anus 297

Measles, prevalence of the 572

Mead, Dr. on quarantine 120

Medical jurisprudence, on 39, 193

—— and physical intelligence 94, 190, 288, 386, 483, 579

—— publications, list of 96, 195, 388, 484, 580

—— obituary 195

—— reports of cases 275

—— police, on 558

—— controversies, on 256, 420

Medicine, historical sketch of 1

—— on affectation in 253

—— on the practice of 394

—— on the present state of 569

Medicines, on the doses of active 55

Meloe vesicatorius, properties of the 22

Mercury, on the internal exhibition of 192

—— in phthisis pulmonalis, on 324

—— on the diuretic effects of 331

Merriman, Mr. on a case of midwifery 417

Midwifery, a case in 337

—— observations on 417

Missletoe, a specific in epilepsy 17

Morris's, Mr. answer to the anti-vaccinists 29

Morison, Mr. on the Dublin pharmacopœia 540

Moramastix, in answer to a Derbyshire Practitioner 136

—— reply to 309

Mortification of the uterus, on 344

—— use of blisters in checking 443

—— on carbonic gas in 536

Moseley's, Dr. case of hydrophobia 550

Mustard, uses of 368

Musk, its efficacy in tetanus 401

N.

Narcotic poisons, on 19

Navy surgeons defended 58

Nephritic complaints, remedy in 458

New York, account of the pestilence at 121

Nitric acid, its use in hepatitis 301

O.

Obituary, medical 195

Ockow, a Chinese remedy for the breast 176

Œsophagus, on the application of lunar caustic to strictures in the 380

—— case of schirrhous ulceration in 475

Oil cake, how made 366

Oil in hydrophobia, on 402

Okes's, Mr. account of a new styptic 199

Old Bailey, black sessions at the 89

Ophthalmia, observations on 13, 474

—— queries concerning 493

Opium in hydrophobia, on 401

—— its efficacy in convulsions 95

Os externum, efficacy of bleeding in rigidity of the 337

Osborn, Dr. on parturition, remarks on 450, 545

Oxford, black assizes at 89

P

Palsy, use of horse-radish in 272

Paralysis, on galvanism in 281

—— case of 154

Paralyfis of the hands, mode of treating 20
Paris, exceffive heat and complaints at 288
Patterfon, Dr. reply to 213
Parifh libraries, plan for 194
Parturition, on bleeding in difficult 337
——— on the neceffity of pain in human 450, 545
Pemberton, Dr. on difeafes of the abdominal vifcera 19
Pemphigus, cafe of 43
Percival, Dr. on the roots of the geum urbanum 436
——— on chorea fancti viti 218
Pharmacopœia, on the Dublin 540
Pharynx, cafe of fcirrhous ulceration of the 475
Phofphoric light from various fubftances 289
Phthifis pulmonalis, obfervations on 9
——— on digitalis in 172
——— prevalence of 289
——— on mercury in 324
Phyficians, report of the Royal College of 97
Phyfick, Dr. on blifters in mortification 443
Piles, remedy for the 171
Pituitous afthma, remedy in the 271
Placenta, on the management of the 281
Plants, botanical defcription of Britifh 169, 270, 363, 457
——— method of rearing tender 386
Pleuritis, cafe of 392
Poifons, obfervations on morbid 82
Pope, Mr. on the treatment of gout 52
——— his cafe of violent cough 54
——— reply to 240
Popliteal aneurifm, cafe of 475
Poppies, effect of exhalation from 288
Portugal, warm fprings in 289
Powder, account of a new ftyptic 199
Prior's, Mr. anfwer to Mr. Chalmers 56
Puerperal convulfions, cafe of 353
Pulmonary confumption, effects of labour in the cure of 448
Pyrofis, effects of oxyd of bifmuth in 281

Q.

Quackery, obfervations on 3, 286

——— inftance of 48

Quarantines, oration on 111

——— obfervations on 423, 497

Queries refpecting ophthalmia 493

Quickfilver, experiments with frozen 289

Quince feeds, ufe of in ophthalmia 15

R.

Rabies, on the fymptoms and cure of 400
Rape oil, whence extracted 366
Redowfki, M. his botanical tour 191
Reid, Dr. on confumption 11
Reform, on medical 3, 185
Refrigeration in fever, on 198
Report of the College of Phyficians 97
Refufcitation of a ftill-born child 307
Refpiration, on difordered 476
Rheumatifm, ufes of horfe-radifh in 272
——— on chronic 275
——— cafe of fever from 362
——— on acute 428
——— cafes of chronic 430
——— cured by mercury 279
——— ufe of arfenic in 565
Ring's, Mr. cafe of tic douloureux 135
——— anfwer to Mr. Golding 137
——— on vaccination 139
——— on burns and fcalds 149
——— anfwer to Mr. Marfon 150
——— cafes of fecondary fmall-pox 314
——— reply to Mr. Davies 315
——— Rowland for an Oliver 386
Roberton, Mr. on the glanders, farcy, and greafe 553
——— on medical police 558
——— report of difeafes in Edinburgh 565
Rouffeau, Dr. on the refufcitation of a ftill-born child 307
Rowley's Dr. cafes confuted 139
Royfton's, Mr. hiftory of medicine 1
Rubeola, cafe of 564
Rupture fociety, report of the new 33
——— on, the utility of 192
Rupture of the aorta, cafe of 391
——— of the liver *ib.*
Ruffia, inftances of longevity in 193
——— progrefs of vaccination in 28

S.

Sabatier's, M. cafes of fractured fternum 200
Saffron, effects of the exhalation of 288
Saunders's, Mr. anatomy of the ear, account of
Sayre's, Dr. cafes of worms
Sage, M. on exhalations from vegetables 288
Scammell's, Mr. cafe of fracture 129
Scalds, on the treatment of 67, 194 206, 238, 360, 405
Scarlatina anginofa, obfervations on 483
Scarpa, profeffor, on difeafes of the eye 15
Scrophulous cafes, remedy for 172
Scurvy grafs, its virtues 270
Seetang, a marine plant, its ufe 191
Shaw's Mr. remedy for ophthalmia 15
——— Dr. cafe of a wound in the lungs 333
——— on tobacco bougies 359

Shearly's Mr. cafes of fungus hæmatodes 391
Sivvens, account of 82
Small-pox in the fœtus, cafes of 177
——— its ravages in Arabia 192
——— obfervations on 480
——— cafes of fecondary 314
——— on inoculation for the 428
———, its prevalence at Edinburgh 566
Smith's Dr. remarks on T. Y. 159
——— cafe of hæmoptyfis 320
Smyth, Mr. on metalic bougies 243
Soporific fever, account of 288
Sour crout, how made 367
Spence, Dr. on fugar of lead in epilepfy 355
Spinal marrow, cafe of inflammation of the 571
Sternum, cafes of fractured 200
——— on fracture of the 415
Stomach, on difeafes of the 18
Stone, Dr. on difeafes of the ftomach 18
Stringham, Mr. on the diuretic effects of mercury 331
Styptic-powder, account of a new 199
Sydenham, on the errors of 86
Sugar of lead, its efficacy in epilepfy 355
Swine, cure for the fcab in 172
Synochus, cafe of 285
Syphilis, on difeafes refembling 82

T.

Tar, medicinal virtues of 460
Taunton, account of the black affize at 89
Teeth, on filling and extracting 210, 553
Tetanus, new method of treating 94
———, efficacy of mufk in 401
———, cafe of 436
Thomas's St. hofpital, lectures at 290
Throat, remedy for ulcers in the 364
Tic douloureux, cafe of 135
——— efficacy of mercury in 176
Tinea capitis cured by vaccination 340
Tobacco bougies, ufes of 350
——— its ufe in rabies 401
Tomlinfon's Mr. cafe of femoral aneurifm 508
Tooth, defcription of an enormous 95
Tumours, on opening 52
——— benefits of digitalis in fcrophulous 172
Typhus, cafes of 92

U.

Ulcers, an injection for finuous 170
——— in the throat, remedy for 364
Ulcus gangrenofum, remedy againft 199
Upton, Mr. on rubeola 564
Uterine hæmorrhage, obfervations on the 281
Uterus, on mortification of the 344
Uva urfi, its ufe in ulcerated lungs 10
Urethra, cafes of ftricture in the 350
——— on lunar cauftic in ftrictures of the 380

V.

Vaccination, review of the controverfy on 23
——— Mr. Marfon, on 70
——— Report of the royal college of phyficians on 97
——— Mr. Ring, on 139
——— tinea capitis cured by 340
Vagina, cafe of worms in the 379
Valentin, Dr. on remunerating Dr. Jenner 341
Vauquelin's M. analyfis of ores 193
Vegetables, on exhalations from 288
Vienna, average of deaths at 28
Village libraries, plan for 194
Vinegar, its ufe in rabies 402

W.

Ward, Mr. reply to 258

Wardrop's Mr. cafes of diffection 577

Warnock's Mr. cafe of fracture 309

Waterhoufe, Dr. his exertions in fpreading vaccination 29

Watfon, Mr. on mercury in phthifis pulmonalis 324

Wawn, Mr. on filling and extracting teeth 210

Weaver, Mr. on cancer and fungus hæmatodes 534

——— on carbonic gas in mortification 536

Weft Indies, on difeafes of the 124

Wildwood ufed by the ancient Britons 173

Willan, Dr. on the cow-pox 27

——— on afthenia 189

Wilfon, Dr. on the nature of fever 462

Winterbottom's Dr. cafe of pemphigus 43

Woodville, Dr. introduced vaccination into France 28

Worcefter, refolutions of the medical practitioners of 194

Worms, on the expulfion of 227

Y.

Yellow fever, obfervations on the 41

——— on the origin of 121

——— on the nature of the 468

Yew tree, defcription and ufes of the 459

